# Statistical Report of the Principal Operations Performed in the London Hospitals during December, 1853

**Published:** 1853

**Authors:** 


*Clinics.*—Statistical Report of the principal Operations performed in the London Hospitals during the Month of December, 1853.

*Lithotomy.*—The three cases reported last month have since recovered. Two operations have been performed during the month. The first was by Mr. Le Gros Clarke on a boy in St. Thomas' Hospital. The patient was six years old, and in good health; a mulberry calculus was extracted by the lateral method, and he recovered very quickly. The second was by Mr. Moore, in the Middlesex Hospital, on a healthy boy from the country, aged seven. He left the Hospital well four weeks afterwards.

*Herniotomy.*—All the cases previously reported have recovered. Number of operations during the month, 9; of which 5 have recovered, 1 is in danger, and 3 are dead. Several of these were cases of great interest. Case 1 was a femoral hernia in a woman, aged 53, under Mr. Stanley's care, in St. Bartholomew's; strangulation 96 hours; taxis scarcely used; sac not opened; recovered well without special after-treatment. Case 2.—An elderly woman, also under Mr. Stanley's care; strangulation 33 hours; taxis had been much abused before admission, sac not opened; the symptoms of peritonitis which came on afterwards were subdued by opium, and she recovered well. Case 3.—A man, aged 24, in Guy's, under Mr. Hilton's care; tumor very large; strangulation nearly 4 hours; sac not opened: recovered well. Case 4.—A woman, aged 50, under Mr. South's care, in St. Thomas'; strangulation one week; sac opened; the gut burst during the operation, and fecal matter escaped; death six hours afterwards; acute peritonitis was found; only a part of the caliber of the gut had been constricted; its coats were ulcerated through at the seat of stricture. Case 5.—A man, aged 30, under Mr Simon's care, in St. Thomas Hospital; strangulation 36 hours; sac opened; a large quantity of universally adherent omentum was found, and concealed it in a small knuckle of intestine; the operation involved a very careful and protracted dissection. Tympanitis came on afterwards, and death on the second day resulted. At the *post mortem*, only the indications of commencing peritonitis were found. Case 6.—A man, aged 43, admitted almost moribund into the Middlesex Hospital, under the care of Mr. Shaw; strangulation four days; much taxis; sac opened; bowel of deep red color, and ecchymosed, but not gangrenous; reduced, but some omentum left in the sac; the omentum sloughed, and on its separation fecal matter escaped; the man has had no peritonitis, and is doing as well as can be expected. Case 7.—A man, aged 40, under Mr. De Morgan's care in the Middlesex Hospital; a large scrotal tumor; strangulation 18 hours; sac opened; nothing but omentum found in sac; it was ligatured and cut away; the man has recovered well. In this case, although no intestine was involved in the hernia, yet the symptoms of strangulation had been well marked. Case 8.—A woman, aged 60, under the care of Mr. Wordsworth in the London Hospital; femoral; strangulation 40 hours; sac opened; favorable progress under free use of stimulants. Case 9.—A woman of middle age, under the care of Mr. Lloyd in St. Bartholomew's Hospital; strangulation four days; sac opened; intestine gangrenous; the intestine was opened and left in the wound, the stricture having been divided over it; she lingered several days in a precarious condition, and then sank.

*Trephining of the skull.*—A man was admitted, insensible, into the London Hospital, under the care of Mr. Adams, having received a violent blow over the right temple. There was great swelling, but no wound. At first, the symptoms were those of concussion only, but twenty hours afterwards an epileptiform fit occurred, after which, well-marked indications of compression set in.—Venesection to zxxx. having been practiced without relief, Mr. Adams cut through the scalp to expose the seat of injury. A fissure having been found extending in such a direction as to cross the course of the middle meningeal artery, the trephine was applied, and some bone having been removed, a large clot was found between the dura mater and cranium. The dura mater was not injured, and the clot having been with some difficulty removed, the symptoms of compression disappeared. On the following day, and throughout the next week, the man was perfectly sensible, though somewhat drowsy. On the ninth day erysipelas occurred, with rigors, and a relapse into a state of coma. The dura mater being tense, and bulging into the wound, Mr. Adams punctured it, and let out a large quantity of serous fluid, without, however, any relief.—Death took place on the fourteenth day, and the autopsy showed acute and general meningitis.

*Ligature of Arteries.*—A case is in St. George's, under care of Mr. Johnson, in which the femoral has been tied on account of a punctured wound. The operation was necessitated by recurring hemorrhage eighty hours after the accident. The man is doing well; we shall probably publish the details of his case at a future time. A second case is in St. Bartholomew's, under the care of Mr. Lawrence, in which a young man was admitted six weeks after having received a deep wound between the first and second metacarpal bones. The bleeding had broken out again four days before admission, and continued afterwards to recur. Mr. Lawrence cut down and tied both ends of the radial artery at the wounded part. The man is doing well. By Mr. Lloyd, in St. Bartholomew's Hospital. Ligature of the radial artery, above and below a wound which had been inflicted by a piece of glass. The patient has done well.

*Aneurism.*—Mr. Hilton's case of popliteal aneurism, in which compression is being pursued, remains under treatment, and almost *in statu quo.* (During the present month two cases of popliteal aneurism have been admitted into St. Mary's, and are now being treated by compression.)

*Amputations.*—The cases reported last month, including the one in which phlebitis was suspected, may be considered convalescent. There have been performed, during the month, 13 amputations—the subjects of which are, 10 of them doing well, and 3 dead. Of these, five were amputations at the thigh for diseased knee-joint, by Messrs. Lawrence, Fergusson, Hawkins, Tatum, and Simon. Mr. Fergusson's patient, a young man, did well at first, but, after a time, was attacked by phlebitis, and died on the twenty-first day. The femoral vein was found full of pus, and there was a small abscess in one lung. Mr. Hawkins' patient died apparently from exhausation, a week after the amputation. The other three cases are recovered. Two cases of primary amputation of the leg, both performed by Mr. Ure in St. Mary's Hospital; one patient, an old man of 63, died from the shock of the severe accident he had sustained; the other is under care. One case of secondary amputation of the leg, recovered. One case of primary amputation of the arm, above the insertion of the deltoid, by Mr. Simon; the man has recovered well. Two cases of amputation at the ankle-joint, for diseased tarsus and for compound fracture; one by Mr. Fergusson, in King's, the other by Mr. Ure, in St. Mary's; both are doing well. One of Mr. Chopart's amputation, for diseased tarsus, by Mr. De Morgan, in the Middlesex; and another of amputation at the metatarso-phalangeal joint, on account of compound fracture, by Mr. Lawrence, in St. Bartholomew's; both recovered. A man, whose leg had been amputated by Mr. Lloyd in St. Bartholomew's Hospital, and whose stump was so nearly healed that we had considered him convalescent, has died within the last week from prostatic abscess and gangrene of the penis. The cause of death had thus no connection with the operation.

*Excision of bones, joints, etc.*—The two cases of excision of the wrist-joint, that of the head of the femur, and those also of parts of the tarsus, previously reported, all remain under care. In Mr. Fergusson's wrist case, some more portions of the carious carpal bones have been removed. In Guy's Hospital, Mr. Cock has operated, during the month, on two cases of diseased tarsus, removing large portions of bone; the patients are progressing favorably. In St. Thomas', Mr. Clarke has performed the successful excision of a part of the upper maxillia on account of a malignant growth from the gum which adhered to the bone. Mr. Simon has removed the whole of a carious metacarpal bone of the thumb from a man who had suffered from disease about the part for five years. Mr. Partridge's case, of somewhat similar character, as also Mr. Hilton's, are still under treatment.

*Removal of Necrosed Bone.*—Eleven operations for the removal of dead bone (the long bones) have been performed during the month, and, together with seven of those previously reported, their subjects continue under care.

*Excision of Malignant Growths.*—Four out of the six cases reported last month have recovered; the other two are doing favorably. During the month, one case of scirrhus of the mammary gland has been operated on, with favorable result. One case of epithelial cancer of the prepuce. The patient in the latter was under the care of Mr. Moore, in the Middlesex Hospital. He was 65 years old, and had perceived some induration of the prepuce for nearly a year, but there had existed a distinct tumor for only about a month. The glands in the groin were not indurated, and, the disease being quite in an early stage, the case was one very favorable for operation. The whole penis was amputated, and the wound soon healed. One case of very extensive epithelial cancer of the lower lip and integument of the chin, operated on in Guy's Hospital, by Mr. Hilton. The portion removed was very large, and, to close the wound, the adjacent skin had to be dissected up and united by means of harelip pins. The result was very successful, union by first intention occurring in almost the whole extent. A case of epithelial cancer of the lip, operated on by Mr. Lloyd, in St. Bartholomew's Hospital, remains under care. In University College Hospital, Mr. Quain has, in one case, extirpated the eyeball, on accoun. of a tumor in the orbit, which, from its progress, had appeared to be malignant; under the microscope, however, it displayed only fibro-plastic characters. In St. Thomas' Hospital, Mr. Simon excised part of the tongue of an elderly man, for a very suspicious-looking growth. Mr. Simon was afterwards, from the result of microscopic examination, inclined to believe that it was warty, and not really cancerous.

*Excision of Non-Malignant Tumors.*—With three exceptions, the cases previously reported are recovered. Seven operations have been performed during the month, all of them successfully. Two were for fibro-cutaneous outgrowths from the labia; two for fatty tumors on the shoulder; one for epulis; one for sero-cystic tumor of the breast; one for an exostosis, the size of a pigeon's egg, from the border of the bicipital groove of the humerus.

*Puncture of the Bladder.*—This operation has been performed once during the month on account of impassable stricture. The patient is in charge of Mr. Cock, in Guy's Hospital, and remains under treatment.

*Operations for Urethral Stricture.*—The cases reported last month are still under treatment. In one case of impermeable stricture, in which, during the month, urethrotomy was performed, death resulted from subsequent peritonitis. In another, under the care of Mr. De. Morgan, in the Middlesex Hospital, the operation of cutting down on the end of the sound just anterior to the stricture, with the intention of prolonging the incision into the proximal part of the urethra, was performed. Mr. De Morgan was not at the time successful in introducing an instrument, but he stated that he believed nevertheless that the obliterated part of the canal had been divided. The patient has done well, and a catheter has subsequently been passed through the whole urethra. The man had, on a previous occasion, had a similar operation performed in another hospital, and he had also had his bladder punctured by the rectum.

*Paracentesis Thoracis.*—The case in St. George's Hospital remains under treatment, and the operation has again been performed.

*Paracentesis Abdominis.*—In *Ascites*, four times, in all with relief. *Ovarian Dropsy* five times; one of the patients died of exhaustion afterwards; there was found at the autopsy a collection of multilocular cysts, in one of which suppuration was commencing.

*Ligature, etc., of Nævus.*—Operations for the cure of nævus have been performed in six cases. In one, under the care of Mr. Cock in Guy's Hospital, the injection of the solution of perchloride of iron has been practised a second time, with the effect of rendering the tumor solid, and without any evil consequence. We leave the others, several of them of peculiar interest, for a future detailed report.

*Fistula in Ano.*—Four cases remain under treatment, and five operations have been performed during the month; all are doing favorably. In one, under the care of Mr. Cock in Guy's Hospital, stricture of the rectum also existed, and was divided at the same time that the fistula was laid open.

The two cases of operations for the cure of ununited fracture, in St. Bartholomew's Hospital, are yet under treatment, and the result cannot in either be spoken of with certainty.

*Plastic Operations.*—Mr. Quain's case of rhinoplasty has, after a small second operation, been discharged well. The other cases previously reported remain under care. Mr. Paget informs us, respecting the case on which three months ago he operated for a large urethral fistula, and apparently without success, that since the man left the hospital, the process of healing has gone on with so much of contraction, that the aperture is now entirely closed, and the man can at times pass urine through the whole length of the canal. Three cases of single harelip have been successfully operated on. In a case of cut throat, in which the wound remained open, Mr. De Morgan, in the Middlesex Hospital, performed the operation of paring the edges, and then uniting them by sutures; it has only partially succeeded.

Mr. Erichsen has successfully treated a case of varicose veins of the leg in a woman, in University College Hospital, by the needle and twisted suture. No inconvenience attended the measure.

The cases of employment of galvanic cautery remain under care.

In King's College Hospital, on a patient who suffered from severe neuralgia, unrelieved by ordinary means, Mr. Bowman performed subcutaneous division of the trunks of the infra-orbital and of the mental branch of the inferior dental nerve. Very great relief has been afforded.

In operations for Cataract no extractions have been performed at the general hospitals during the month. The two cases undergoing treatment by absorption remain under care.

Medical Times and Gazette, January 21, 1854

